# Viral Induced Protein Aggregation: A Mechanism of Immune Evasion

**DOI:** 10.3390/ijms22179624

**Published:** 2021-09-06

**Authors:** Elena Muscolino, Laura-Marie Luoto, Wolfram Brune

**Affiliations:** 1 Leibniz Institute for Experimental Virology (HPI), 20251 Hamburg, Germany; elena.muscolino@upf.edu (E.M.); laura.luoto@leibniz-hpi.de (L.-M.L.); 2 Molecular Virology Group, Department of Experimental and Health Sciences, Universitat Pompeu Fabra, 08003 Barcelona, Spain

**Keywords:** Murine Cytomegalovirus (MCMV), Herpes Simplex Virus (HSV), Epstein-Barr Virus (EBV), baculovirus, M45, ICP6, BORF2, ORF61, VPS26B, TBC1D5

## Abstract

Various intrinsic and extrinsic factors can interfere with the process of protein folding, resulting in protein aggregates. Usually, cells prevent the formation of aggregates or degrade them to prevent the cytotoxic effects they may cause. However, during viral infection, the formation of aggregates may serve as a cellular defense mechanism. On the other hand, some viruses are able to exploit the process of aggregate formation and removal to promote their replication or evade the immune response. This review article summarizes the process of cellular protein aggregation and gives examples of how different viruses exploit it. Particular emphasis is placed on the ribonucleotide reductases of herpesviruses and how their additional non-canonical functions in viral immune evasion are closely linked to protein aggregation.

## 1. Introduction

Proteins obtain their functional conformation through correct folding of the primary amino acid sequence into a three-dimensional structure. However, the folding process can be disrupted by genetic mutations, incomplete translation, environmental stress factors, or a lack of ribosomal quality control, among other factors [1]. In such cases, or if the folding capacity of the cellular chaperone machinery is exceeded, for instance due to massive production of viral proteins during infection, misfolded proteins with incorrectly exposed hydrophobic surfaces start to accumulate and form aggregates. Protein aggregates can either be amorphous or they can form structurally ordered oligomers or amyloid fibrils [2]. Although aggregated conformation is a prerequisite for the functionality of some proteins, accumulation of protein aggregates is usually a cytotoxic process or even a pathological hallmark of disease, the latter being observed for instance in systemic amyloidosis [3] or neurodegenerative disorders such as Alzheimer’s disease and Parkinson’s disease [4]. To prevent cytotoxic effects accompanying inappropriate protein aggregation, cells target aggregated proteins for degradation via the ubiquitin-proteasome pathway, chaperone-mediated autophagy, or aggrephagy [1].

Protein aggregation during viral infection can either be a process actively induced by the virus or an indirect consequence of perturbed cellular proteostasis and remodeling of the cellular architecture taking place during infection. In this review, we summarize the key steps of protein aggregate formation and clearance with a special focus on how viruses as intracellular pathogens exploit these cellular pathways, usually by playing a role in cell survival and innate immune responses. We provide examples of how viruses across several viral families make use of protein aggregation for efficient progeny production, immune evasion, and enhanced virulence. Thereby we especially highlight the non-canonical role of the herpesviral ribonucleotide reductase in counteracting cellular immune defenses.

## 2. Protein Synthesis and Folding

Viral infection initiates a series of stress responses leading to the compartmentalization of RNA and proteins. RNA is compartmentalized into membraneless bodies, known as RNP granules, that are distributed both in the cytoplasm and the nucleus [5]. The formation of RNP granules is a dynamic process with the aim of concentrating specific cellular components upon stress. Stress granules and P-bodies are two types of granules present in the cytoplasm that are involved in RNA silencing and decay [6,7]. Stress granules form after mRNA translation has stalled, and they are associated with several RNA-interacting proteins, translation initiation factors, and other proteins. P-bodies, on the other hand, contain mRNAs associated with the mRNA silencing and decay machinery [8]. Stress granules and P-bodies are found in close proximity to each other, suggesting an exchange of components between them [7]. Formation of RNP granules occurs frequently upon viral infection and is a dynamic event that can cause a redistribution of mRNA and translation arrest, affect mRNA localization, and eventually induce autophagy and other antiviral responses [9]. The formation of stress granules during viral infection and how viruses affect them have been reviewed elsewhere [10]. As membrane-less compartments, it has been proposed that they arise through a process called liquid-liquid phase separation (LLPS), a liquid state of matter that can rapidly and reversibly change [8,11]. This process is mediated by intrinsically disordered proteins with low complexity domains that are found to be present in RNP granules [12].

Unlike RNP granules, protein aggregates are often associated with membranes and are characterized by a state of matter that does not change reversibly [13,14,15]. Aggregates can be detected with different methods including the use of dyes, fractionation techniques, or advanced microscopy techniques (Table 1).

As aggregate accumulation has cytotoxic effects, cells aim to either prevent aggregation or to direct aggregates towards degradation. To understand how protein aggregates are formed, we will briefly recapitulate the principles of protein folding. Upon their synthesis on ribosomes, proteins must fold into a three-dimensional structure to fulfill their biological function [22]. Folding into the tertiary protein structure is energetically unfavorable and, if unsuccessful, can lead to the accumulation of misfolded proteins with exposed hydrophobic domains. In the endoplasmic reticulum (ER), proteins that do not fold properly are targeted for ER-associated degradation (ERAD), which involves the proteasome [23]. Protein translocation, folding, and degradation are facilitated by molecular chaperones. There are three major groups of ER chaperones that can localize at the nascent chain: (i) heat shock proteins (e.g., Hsp70, Hsp40, and Hsp90) and several co-chaperones (e.g., CDC37 and the BAG3) that promote protein folding and prevent aggregation; (ii) ER lectins (e.g., Calnexin, calreticulin) that keep immature and unfolded proteins in the ER; (iii) thiol oxidoreductases (e.g., PDI) that are involved in the formation of disulfide bonds [24]. Interestingly, the same heat shock proteins that are involved in protein folding also mediate the degradation of misfolded proteins [25,26].

Various stress conditions, including viral infection, can lead to the accumulation of unfolded or misfolded proteins in the ER and induce ER stress, which in turn activates the unfolded protein response (UPR) [27]. The UPR consists of three ER-to-nucleus signaling pathways that collectively work to restore homeostasis by reducing translation, increasing the expression of chaperones and foldases, and upregulating ERAD [28,29]. Accumulation of damaged proteins can disrupt cellular homeostasis and lead to aging, degenerative diseases, and even cell death. Examples are the accumulation of protein aggregates in neurodegenerative disorders such as Alzheimer’s disease, Parkinson’s disease, and Huntington’s disease [30]. Thus, damaged or misfolded proteins must be repaired or eliminated by protein quality control systems consisting of the aforementioned molecular chaperones or proteolytic systems. Chaperones are mainly, but not exclusively, involved in repairing damaged proteins, while irreparably damaged proteins are identified and selected for degradation.

## 3. Disposal of Protein Aggregates

**Figure 1:**
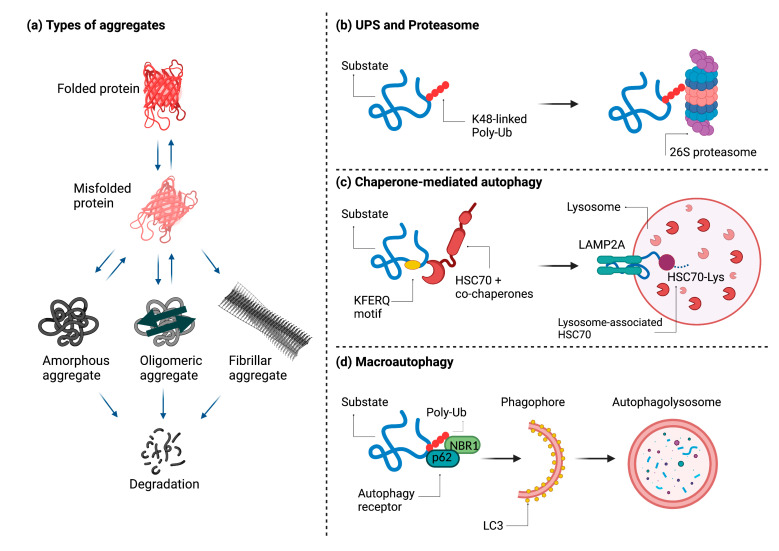
Types of aggregates and degradation mechanisms. A functional protein is normally found in a properly folded three-dimensional state. Aggregation, which is normally prevented by molecular chaperones, can occur as a result of different stimuli and lead to the formation of three different types of aggregates: amorphous, oligomeric, or fibrillar. Except in the case of fibrillar aggregates, these proteins can return to their folded state. If this does not happen, they are degraded (**a**). Aggregates can be degraded in three different ways: by the ubiquitin-proteasome system (**b**), by chaperone-mediated autophagy (**c**), or by aggrephagy, a special form of macroautophagy (**d**). Figure created with BioRender.com (accessed on 3 August 2021).

To avoid cytotoxic effects, cells target accumulating protein aggregates for degradation. The highly regulated process of aggregate disposal can take place either through the ubiquitin-proteasome system (UPS), through chaperone-mediated autophagy (CMA), or through macroautophagy, which is also called aggrephagy when the substrate is an aggregate (Figure 1) [1,31,32].

The UPS involves a series of enzymes, E1 (ubiquitin-activating enzyme), E2 (ubiquitin-conjugating enzyme), and E3 (ubiquitin ligase), that mark the polypeptide with ubiquitin residues targeting it for degradation through the proteasome [33,34]. Ubiquitinated substrates are subsequently transferred to the proteasome and degraded. It should be noted that aggregates degraded by autophagy can also be ubiquitinated [35]. CMA is a multi-step process that takes place in the cytosol and is regulated by Hsp70 and several co-chaperones [36]. The first step involves substrate recognition through a KFERQ-like motif that is present in all CMA substrates and becomes accessible to Hsc70 when the target protein is misfolded [37]. The substrate is subsequently targeted to lysosomes through the lysosome-associated membrane protein type 2A (LAMP-2A) receptor [38]. Here the protein unfolds, crosses the lysosomal membrane, and undergoes complete degradation as soon as it reaches the lysosomal matrix.

The term aggrephagy, which was introduced by Per Seglen, describes the selective sequestration and degradation of protein aggregates by autophagy [39]. UPS and CMA are only capable of degrading one extended polypeptide at a time. When the accumulation of unfolded proteins exceeds the capacity of these two systems, misfolded proteins are actively transported along microtubules to pericentriolar inclusions called aggresomes [40]. The aggresomes accumulate around microtubule-organizing centers (MTOCs). However, the mechanism of active aggregate transport to the MTOCs remains incompletely understood [41]. An active role in this process has been attested to Parkin-mediated K63-linked polyubiquitination as a signal coupling misfolded proteins to the dynein motor complex via the adaptor protein histone deacetylase 6 (HDAC6) [42,43,44,45]. Misfolded proteins are thereby sequestered in aggresomes and can be subsequently cleared by autophagy.

The aggresome itself is an insoluble and metabolically stable structure surrounded by vimentin and keratin filaments and usually contains ubiquitinated proteins. Autophagy adaptors such as p62, NBR1, and autophagy-linked FYVE protein (ALFY) are also present in aggregates and can be involved both in their formation and autophagy-mediated degradation [46]. Two other types of described aggresome-like structures that do not localize at the MTOC include the insoluble protein deposit (IPOD) and the juxtanuclear quality control compartment (JUNQ). IPOD does not contain ubiquitinated proteins and is located at the cell periphery, whereas JUNQ harbors ubiquitinated proteins and is located close to the nucleus. However, the relationship between these two structures and aggresomes has not yet been fully elucidated [47].

## 4. Aggregate Formation during Viral Infection

Viral infection causes profound changes in the cell architecture, for instance by inducing the formation of aggregates or insoluble inclusion bodies that contain viral structural proteins [48,49]. Although cells usually rapidly dispose of such potentially cytotoxic structures to ensure survival, several viruses are known to exploit the aggregation process for enhanced replication, immune evasion, and virulence. Large cytoplasmic DNA viruses make use of aggresome-like viral factories as scaffolds to facilitate replication and assembly [49,50,51], whereas, for instance, adeno- and herpesviruses promote aggregation of cellular immune effectors and signal transducers to suppress their antiviral effector or signaling functions [52,53,54] (Table 2).

Occasionally, an aggregated conformation constitutes a prerequisite for a fully functional virulence factor, as observed in highly virulent strains of influenza A virus [55].

African swine fever virus (ASFV), a member of the *Asfarviridae* family, is an example of a large DNA virus that forms cytoplasmic viral factories resembling cellular aggresomes as a means of concentrating viral DNA and essential structural components for progeny virion production [50]. While aggresomes localize directly to MTOCs, ASFV viral factories form in their vicinity. Both structures are surrounded by a vimentin cage and recruit mitochondria and cellular chaperones, such as Hsp70. Similar to aggresomes, the structural integrity of ASFV viral factories is dependent on an intact microtubule network, as evidenced by dispersal of the compact perinuclear factories upon treatment with the microtubule-depolymerizing drug nocodazole. The authors proposed that, following cell entry, ASFV nucleocapsids are recognized as aggregates by the aggresome machinery and transported along microtubules to the MTOC. The vimentin cage prevents the distribution of capsids throughout the cytoplasm. Since the capsids already contain enzymes necessary for viral genome replication, the nascent aggresomes serve as ideally shielded intracellular sites for localized production of viral DNA.

Baculoviruses, which infect several orders of insects and form characteristic polyhedric or oval occlusion bodies depending on the virus species, have also been associated with protein aggregation. The baculoviral protein polyhedrin is originally known as a structural component of occlusion bodies in which the virions are immobilized in a crystalline protein lattice, allowing them to persist outside the host and remain infectious even under harsh environmental conditions [56]. Guo et al. have investigated the structural nature of *Bombyx mori* nucleopolyhedrovirus (BmNPV) polyhedrin during infection and could show that BmNPV polyhedrin shares characteristics of aggresomes at early times post-infection, including co-localization with both the MTOC marker γ-tubulin and aggresome-targeted fusion protein GFP-250, microtubule-dependent formation, as well as recruitment of mitochondria and Hsc/Hsp70 [57]. The authors conclude that since polyhedrin is produced in large quantities within a short time during baculovirus infection, the protein is dependent on the cellular chaperone machinery for correct folding, and the observed aggresomes potentially serve polyhedrin storage until it is processed by the chaperones. As the infection proceeds, polyhedrin recovers its natural conformation and co-localizes with the autophagosomal marker LC3 in the phagophore of autophagosomes. Indeed, polyhedrin protein levels and the production of BmNPV occlusion bodies decrease significantly upon chemical inhibition of autophagy with 3-methyladenine [57].

During adenovirus type 5 (Ad5) infection, large cytoplasmic structures fulfilling the criteria for aggresomes are induced by the viral early proteins E4-11k and E1B-55k. Both proteins redistribute and sequester members of the cellular Mre11/Rad50/Nbs1 (MRN) DNA repair complex from the nucleus to distinct perinuclear accumulations to prevent DNA damage response and concatenation of viral DNA [52,53]. In Ad5-transformed cells, p53 also colocalizes in the perinuclear bodies [58], which are surrounded by a vimentin cage, stain with the MTOC marker γ-tubulin and require an intact microtubule-based cytoskeleton for their formation as they drastically reduce in number upon nocodazole treatment. In the absence of E4-11k, Mre11, Rad50, and Nbs1 are mostly present in the soluble cell fraction but accumulate in the insoluble fraction in E4-11k-transfected cells, indicating that E4-11k expression reduces the solubility of the MRN complex constituents [52]. Apart from the MRN proteins, several components of RNA-processing P-bodies are relocalized to E4-11k-containing aggresomes [59]. However, most of these proteins are also present in aggresomes induced by chemical stress, independent of E4-11k, except for the P-body component RNA helicase Ddx6. Based on the role of E4-11k in stimulating viral late gene expression via viral late mRNA accumulation, Greer et al. postulated that E4-11k sequesters Ddx6 in aggresomes to perturb P-body function and thereby the decay of viral mRNAs, which in turn stimulates viral late gene expression. All in all, Ad5 efficiently exploits the cellular aggresome pathway to redistribute, sequester, and finally degrade several host proteins for immune evasion and enhanced viral replication [59].

Another viral protein with a propensity to form aggregates is the influenza A virus (IAV) accessory protein PB1-F2. It is translated from an alternative reading frame in the PB1 polymerase gene and can be classified as an intrinsically disordered protein; it is able to alter between a disordered, an α-helical, and a β-sheet conformation depending on the physicochemical conditions [59]. In a membrane-mimicking environment, PB1-F2 acquires a β-sheet conformation, in which the protein is capable of oligomerizing to amyloid fibers, as determined by dynamic light scattering and electron microscopy. The amyloid β-sheet structures are also detected in wild-type IAV-infected cells but are absent upon ΔPB1-F2-mutant infection. Furthermore, PB1-F2 has been shown to rupture membranes at nanomolar concentrations [55,60] potentially by inducing the formation of nonselective ion channels [61]. However, membrane lysis is only observed in the presence of amyloid-like PB1-F2 oligomers and fragmented fibers, instead of amorphous PB1-F2 aggregates, indicating that the cytotoxicity of PB1-F2 is determined by the protein’s conformational state. In the infection context, wild-type IAV induces membrane rupture at late time points in A549 epithelial and U937 monocyte cells, whereas the effect is less pronounced with a ΔPB1-F2-virus. The role of PB1-F2 in destabilizing membrane integrity is corroborated by electron microscopy showing more membrane fragmentation and damage at late times during wild-type IAV infection compared to a ΔPB1-F2 mutant [55]. As PB1-F2 has been shown to accumulate as amyloid-like oligomers late in infection [62], these very amyloids are likely responsible for the lytic activity of PB1-F2 and the resulting membrane damage during IAV infection [55].

## 5. Herpesvirus Proteins and Aggregate Formation

The herpesviruses (*Herpesviridae*) are a family of double-stranded DNA viruses with large genomes of up to 230 kbp, which can be further subdivided into three subfamilies: *Alphaherpesvirinae*, *Betaherpesvirinae* and *Gammaherpesvirinae*. Human pathogenic herpesviruses include the alphaherpesvirus Herpes simplex virus type 1 (HSV-1), the betaherpesvirus Cytomegalovirus (CMV), the gammaherpesviruses Epstein-Barr virus (EBV), and Kaposi’s sarcoma-associated herpesvirus (KSHV) [63,64].

**Figure 2:**
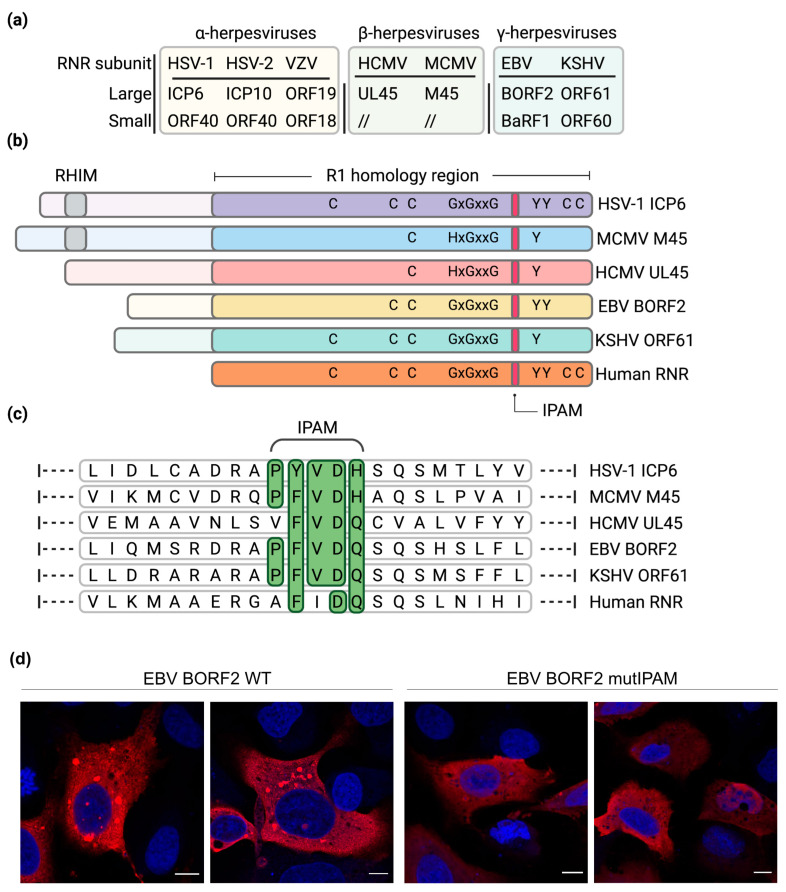
Ribonucleotide reductase in herpesviruses. (**a**) Names of the large (R1) and small (R2) RNR subunit homologs present in alphaherpesviruses, betaherpesviruses, and gammaherpesviruses. (**b**) Schematic representation of human and viral R1 proteins. Residues important for RNR activity are shown. M45 and ICP6 contain a RHIM domain, which is not present in the other viral R1 proteins. (**c**) Alignment of the Induced Protein Aggregation Motif (IPAM). Residues corresponding to the consensus shaded green. (**d**) Requirement of the EBV BORF2 IPAM for the formation of cytoplasmic protein condensates. U2OS cells were transfected with plasmids encoding Flag-tagged wildtype BORF2 or an IPAM mutant. Flag-tagged proteins (red) were detected by indirect immunofluorescence staining and confocal microscopy. Nuclei were counterstained with DAPI (blue). Scale bar, 10 µm. Panels a, b, and c were made with BioRender.com (accessed on 3 August 2021).

Herpesviruses have coevolved with their respective host species during extensive periods of time, giving rise to specific adaptations and ultimately a balance between virus and host defenses. Consequently, most herpesviruses are characterized by a narrow host range. Furthermore, herpesviruses are highly disseminated in nature and almost all examined animal species have been shown to carry at least one herpesvirus species [63]. To ensure productive infection in the hostile cellular environment, herpesviruses have acquired diverse genes with a high degree of homology to cellular genes involved in various processes, such as immune regulation and nucleotide metabolism, probably by hijacking them from the host. To facilitate replication in quiescent and non-dividing cells with restricted dNTP pools, most herpesviruses encode their own ribonucleotide reductase (RNR) enzymes to catalyze the synthesis of dNTPs (Figure 2a,b).

Alphaherpesviruses and gammaherpesviruses encode an active heterotetrameric RNR enzyme consisting of two large (R1) and two small (R2) subunits. In contrast, betaherpesviruses lack an active RNR enzyme and merely harbor the R1 subunit. In addition to their canonical role in nucleotide metabolism, herpesvirus R1 proteins carry out additional functions in viral immune evasion [65].

Although devoid of enzymatic activity, the R1 protein M45 of the murine cytomegalovirus (MCMV) has been shown to be essential for viral replication both in vitro and in vivo. Viruses lacking M45 induce rapid programmed cell death (PCD) in SVEC4-10 endothelial cells and macrophages, thereby precluding viral spread [66,67]. Subsequent studies have shown that M45 inhibits PCD by blocking the receptor-interacting protein kinase (RIPK) 1 and 3 (RIPK3) [68,69,70]. Upon activation by death receptor stimulation or by viral infection, RIPK1 complexes with RIPK3 through a RIP homotypic interacting motif (RHIM) and induces downstream signaling. RIPK3 phosphorylates the mixed lineage kinase domain-like pseudo-kinase (MLKL), leading to cell membrane disruption and induction of necroptosis, a special form of PCD [71,72]. At its N-terminus, the viral M45 protein carries a RHIM motif that blocks the formation of the RIPK1-RIPK3 complex. M45 also inhibits RIPK1-independent induction of necroptosis through interaction with the RHIM of TRIF, RIPK3, and DAI [68,70,73]. Recent studies demonstrated that the RIPK1-RIPK3 signaling platform (called necrosome) forms an amyloid signaling complex characterized by β-sheets that initiate necroptosis [74]. In vitro studies by Pham et al. showed that the N-terminal 90 residues of the M45 protein, which contain the RHIM domain, self-assemble into homo-oligomeric amyloid fibrils and interact with the RHIM domain of RIPK1, RIPK3, and DAI forming heteromeric amyloid fibrils [75]. Whether M45 initiates the formation of such amyloid fibrils in cells remains to be shown.

Similar to M45, the R1 protein ICP6 of the human alphaherpesvirus HSV-1 also contains a RHIM and inhibits RIPK1/RIPK3-dependent necroptosis in human cells [76,77]. Interestingly, ICP6 also suppresses apoptosis, another form of PCD, by binding and blocking caspase-8 [78]. Thus, ICP6 concurrently inhibits two forms of PCD, apoptosis and necroptosis.

Besides inhibiting necroptosis through its RHIM, MCMV M45 also prevents the activation of the proinflammatory transcription factor NF-κB by interacting with the NF-κB essential modulator (NEMO), the regulatory subunit of the Inhibitor of κB Kinase (IKK) complex responsible for NF-κB activation. M45 binds to NEMO and induces its autophagy-mediated degradation [79]. Subsequent work showed that M45 causes the accumulation of RIPK1 and NEMO in detergent-insoluble protein aggregates in the cytoplasm. Aggregate formation is strongly increased when autophagy is blocked (i.e., in autophagy-deficient cells or in the presence of drugs inhibiting the autophagosome-lysosome degradation pathway), indicating that aggregates are degraded by autophagy [54]. The C-terminal part M45 is necessary for the formation of RIPK1 and NEMO-containing aggregates and their degradation. It contains an Induced Protein Aggregation Motif (IPAM) required for M45 self-interaction, for its interaction with RIPK1 and NEMO, and for aggregate formation. In the same way as M45, the R1 homolog of HSV-1, ICP6, also induced the formation of RIPK1-containing protein aggregates and their degradation by aggrephagy [54].

MCMV infection initially leads to a very brief and transient activation of NF-κB. Curiously, this initial activation is mediated by M45 brought into the cell by viral particles (M45 is a constituent of the viral tegument). Shortly thereafter, NEMO is inactivated and degraded, resulting in a complete block of all canonical NF-κB activating pathways [80]. How exactly the initial activation is triggered remains unknown. However, the most plausible hypothesis is that the small quantities of M45 imported through the infecting virions bring RIPK1 and NEMO molecules together in nascent aggregates. These micro-aggregates could function similarly to signaling platforms and induce activation of the RIPK1-IKK-NF-κB signaling pathway. Later, RIPK1 and NEMO become sequestered in larger aggregates and subsequently degraded by aggrephagy, resulting in a complete blockage of the signaling pathway [54].

Recently, Cheng et al. revealed an additional non-canonical function of herpesviral R1 proteins. They reported that the R1 proteins of EBV, KSHV, and HSV-1 play a role in counteracting cellular APOBEC3 (A3) ssDNA cytosine deaminases [81,82]. A3s are known viral restriction factors that counteract several viral families with ssDNA replication intermediates both in a deaminase-dependent and -independent fashion [83]. By catalyzing the deamination of cytosine bases to uracils preferentially on the DNA minus-strand, A3s have the potential to introduce C/G-T/A hypermutations into replicating viral genomes [84], provided that they have access to the site of viral replication. Viruses exposed to the pressure by A3 proteins have in turn evolved countermeasures to protect their genomes from deleterious mutations. In particular, the host-virus arms race between several cytoplasmic A3 members and human immunodeficiency virus type 1 (HIV-1) has been characterized extensively [85,86,87,88].

Herpesviruses, which replicate their DNA in the nucleus, are by default especially susceptible to mutation by nuclear A3 proteins. To protect their genomes from detrimental deamination events during the lytic phase of replication, EBV and HSV-1 have repurposed their respective R1 proteins, BORF2 and ICP6, to relocalize nuclear APOBEC3 proteins A3A and A3B to the cytoplasm, as evidenced by data from Cheng et al. Upon overexpression, BORF2 colocalizes with both A3A and A3B in either elongated or dot-shaped cytoplasmic structures, respectively. Similar cytoplasmic colocalization is observed upon overexpression of the KSHV R1 protein ORF61 with both A3A and A3B [81,82].

Upon induction of EBV lytic replication, the authors first observe BORF2 colocalization with A3B in dot-shaped structures both in the nucleus and the perinuclear space followed by accumulation of cytoplasmic bodies containing both proteins at later times. In imaging studies, these BORF2- and A3B-containing bodies colocalized with BiP/GRP-78 and TRAPα indicate an association with the endoplasmic reticulum. Given that the A3B relocalization is absent upon a ∆BORF2 mutant EBV reactivation, the authors concluded that the process is entirely dependent on BORF2 [82]. In the case of HSV-1, distinct cytoplasmic structures containing A3B and the HSV-1 R1 protein ICP6 were absent in a wildtype HSV-1 infection but appeared during an infection with a ∆ICP4 mutant HSV-1, which is characterized by abnormally high levels of viral immediate-early proteins and ICP6 [81].

In addition to R1 proteins, herpesviral ubiquitin deconjugases (DUBs) have been described to promote aggregate formation [89,90]. DUBs are deubiquitinating cysteine proteases that catalyze the removal of ubiquitin residues from their substrates. In herpesviral genomes, DUBs are encoded in the N-terminal domain of the viral large tegument proteins, and they promote viral replication by interfering with various steps of the host type I interferon (IFN) response [91,92,93]. DUBs encoded by EBV (BPLF1) and KSHV (ORF64) have both been shown to inhibit the ubiquitination of RIG-I [94,95], whereas the HSV-1 (UL36) and HCMV (UL48) counterparts interfere with TRAF3 ubiquitination [96,97]. UL48 additionally interacts with the HCMV R1 protein UL45, and the two proteins cooperatively inhibit NF-κB signaling at late stages of HCMV infection by targeting RIPK1. Similarly, MCMV DUB M48 interacts with both the R1 homolog M45 and RIPK1, indicating a conserved immunomodulatory function of viral DUBs and R1 proteins in betaherpesviruses [98].

Regarding the molecular mechanism behind RIG-I inhibition by EBV BPLF1, Gupta et al. have shown that the catalytic domain of BPLF1 is recruited to a trimolecular complex encompassing TRIM25 ligase and 14-3-3 molecular scaffold proteins [89]. The BPLF1/TRIM25/14-3-3-containing complex does not colocalize with the stress granule marker TIA-1 distinguishing it from viral-induced stress granules. Instead, it represents an aggregated cytoplasmic structure in which autoubiquitinated TRIM25 is sequestered, preventing it from ubiquitinating RIG-I and thereby activating the type I IFN response. Interestingly, the autophagy receptor p62 is present on the aggregated BPLF1/TRIM25/14-3-3-complex, suggesting that TRIM25 might be targeted for degradation by aggrephagy. However, the authors observed stabilization of TRIM25 levels in the presence of catalytically active BPLF1 indicating that the EBV DUB might instead inhibit selective autophagy.

According to Gupta et al., binding of catalytically active BPLF1 to 14-3-3 is essential for TRIM25 aggregate formation, as well as for inactivation of the IFN response. Active BPLF1 also modifies TRIM25 polyubiquitin chains, giving rise to a mono/di-ubiquitinated form with a slower turnover, whereas catalytically inactive BPLF1 induces polyubiquitination and subsequent degradation of TRIM25 without causing protein aggregation [89]. Since the BPLF1 domain required for interaction with 14-3-3 is conserved in functional homologs of other herpesviruses, the authors extended their observations to HSV-1, HCMV, and KSHV DUBs. They discovered that the catalytic domains of HCMV UL48 and KSHV ORF64 share the capacity of EBV BPLF1 to autoubiquitinate and sequester TRIM25 in cytoplasmic aggregates, thereby precluding activation of type I IFN response. In contrast, the HSV-1 UL36 catalytic domain alone neither induces TRIM25 aggregation nor inhibits the IFN response, which the authors attributed to the observed weak interaction between UL36 and 14-3-3 [90].

In summary, herpesviruses possess several proteins that promote the sequestration of diverse host immune effector molecules in protein aggregates. However, the involvement of autophagy in the removal of these aggregates has not been fully elucidated. Whereas M45- and ICP6-induced aggregates containing mediators of programmed cell death are cleared by aggrephagy, the fate and the structural characteristics of the structures encompassing BORF2/ICP6 and A3B remain to be further investigated. It is not excluded that they are protein aggregates, which are subsequently targeted to autophagic clearance. Finally, the potential role of herpesviral DUBs in manipulating the autophagic response awaits further investigation.

## 6. Requirements for Viral Induced Protein Aggregation and Disposal: IPAM and Autophagy Adapters

Viral R1 proteins have acquired additional functions, and some of them are involved in the formation and disposal of protein aggregates. In MCMV M45, a short sequence motif within the C-terminal part of the protein was identified by alanine scanning mutagenesis. The 5-amino acid motif PFVDH, which is required for M45-induced protein aggregation, was named Induced Protein Aggregation Motif (IPAM). It is necessary for M45 oligomerization, as well as for M45 interaction with RIPK1 and NEMO. In the absence of the IPAM, M45 is unable to oligomerize, suggesting that the motif catalyzes the polymerization and aggregation of M45 with its interacting proteins [54]. The fact that a small motif can mediate all these functions is most likely due to the ability of M45 to interact with RIPK1 and NEMO after forming dimers or oligomers. Interestingly, the IPAM appears to be conserved in R1 proteins of related herpesviruses (Figure 2c). The IPAM consensus sequence (P-F/Y-V-D-H/Q) is, for instance, present in HSV-1 ICP6. Indeed, the ICP6 IPAM was shown to be required for interaction with RIPK1, aggregate formation, and inhibition of necroptosis [54]. A motif search revealed that the IPAM consensus motif is present in more than 70 viral R1 homologs, mostly from the herpesviruses but also from baculoviruses and giant viruses (e.g., mimivirus and pandoravirus). This finding suggests a broader conserved function. Of note, the IPAM consensus sequence is not fully conserved in the human cytomegalovirus R1 protein, UL45, which lacks the proline residue of the motif (Figure 2c).

**Figure 3:**
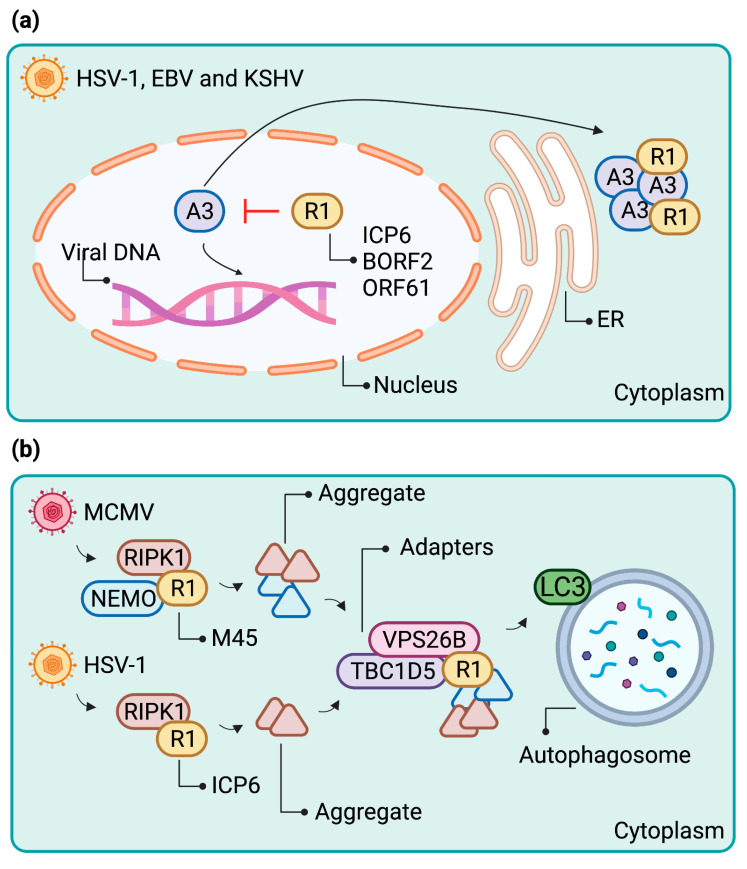
Schematic representation of R1s and aggregate formation. Detailed mechanism of cellular protein degradation by herpesvirus R1s. (**a**) HSV-1, EBV, and KSHV R1 target nuclear APOBECs (A3) and concentrate them to perinuclear clusters that resemble protein aggregates. (**b**) MCMV and HSV-1 R1 induce the formation of aggregates containing antiviral proteins. They then recruit autophagy adaptor proteins that facilitate the removal of aggregates through aggrephagy. Figure created with BioRender.com (accessed on 26 August 2021).

While the IPAM appears to be conserved in several R1 viral homologs, the cellular interaction partners appear to be different. M45 and HSV-1 ICP6 both interact with RIPK1, but whereas M45 also interacts with NEMO, ICP6 interacts with caspase-8 [78]. Whether ICP6 inactivates caspase-8 by inducing its aggregation remains to be tested. ICP6 can also interact with APOBEC3A and, to a lesser extent, with APOBEC3B. ICP6 was shown to promote APOBEC3A accumulation in cytoplasmic condensates [81]. Similarly, the EBV and KSHV R1 proteins, BORF2 and ORF61, respectively, induce APOBEC3B relocation to perinuclear dot-like structures [82] (Figure 3a). Whether these structures represent protein aggregates remains to be investigated. However, preliminary data indicated that that the BORF2 IPAM is required for the formation of the dot-like structures (Figure 2d).

Although the formation of NEMO and RIPK1 aggregates contributes to their loss of function, it is interesting to note that both M45 and ICP6 promote their degradation via autophagy. Two proteins involved in autophagosome targeting of NEMO and RIPK1 aggregates have been identified: the retromer component VPS26B and the LC3-interacting protein TBC1D5. M45 and ICP6 both interact with these autophagy adaptor proteins, and autophagic degradation is impaired in their absence [54] (Figure 3b).

Finally, an IPAM has not only been found in several viral proteins but also in a few cellular proteins. For example, the co-chaperone BAG3 bears a similar motif (PFFVDH). Chaperones such as Hsp70, BAG3, and HSPB8 are involved in protein quality control, and in the presence of aggregates, they form a complex that directs aggregates into autophagosomes for degradation [99]. Since M45-induced aggregates do not co-localize with Hsp70 but only do so with LC3B, the herpesviruses and other large dsDNA viruses may have evolved a mechanism that mimics the function of a cellular chaperone.

## 7. Conclusions and Open Question

We are only beginning to understand how virus-host cell interaction is influenced by the formation and turnover of aggregates. In some cases, the host induces aggregation of certain viral components to prevent viral replication. In other cases, viruses promote the aggregation of host molecules needed for antiviral defense. In particular, herpesviruses appear to induce protein aggregation and promote aggrephagy through a protein present in all herpesviruses and many other large dsDNA viruses, the ribonucleotide reductase R1 homolog. By alanine scanning mutagenesis, a specific motif, called IPAM, was identified, which mediates protein aggregation. This IPAM is conserved in the R1 proteins of many herpesviruses and several other large DNA viruses. It is currently not known whether all proteins with a conserved IPAM can induce aggregate formation or whether additional structural features are required. It is also not known whether mutagenesis of the IPAM affects the catalytic activity of the ribonucleotide reductase.

The disposal of aggregates through aggrephagy appears to be conserved in at least two herpesviruses, MCMV and HSV-1. It should also be mentioned that the viral proteins themselves aggregate and are degraded by autophagy. However, it is unclear whether autophagic activity is upregulated by viral infection or whether the presence of aggregates induces it.

Autophagy adaptors are important mediators of selective autophagy of aggregates. The best-known ones are p62, NBR1, and ALFY [1]. Curiously, MCMV and ICP6 do not use the ‘classical’ adaptors but recruit the retromer component VPS26B and TBC1D5. Are these proteins also involved in aggrephagy in the absence of viral infection, or do the viruses repurpose the two proteins for non-physiological functions? Additionally, do other viruses use them too? These and other unsolved questions should be worth investigating in future studies.

## Figures and Tables

**Table 1:** Selected methods for detection of protein aggregates in cells. The listed methods make use of fluorescent dyes, solubility properties of aggregates, and different microscopy techniques.

Detection Method	Principle	Additional Remarks
Thioflavin-T (ThT) [16]	Fluorescent rotor dye Free rotation inhibited upon intercalation into the β-sheet structure of amyloid fibrils leading to (or significantly increasing existing) fluorescence emission.	Emission maximum at ~485 nm overlaps with the intrinsic fluorescence of some cellular constituents [17]
ProteoStat^®^ [18]		High signal intensity Emission maximum at 600 nm enables the use of additional fluorescent markers Suitable for high-throughput screening by flow cytometry
BSB (trans, trans)-1- bromo-2, 5-bis-(3-hydroxycarbonyl-4-hydroxy) styrylbenzene [19]	Fluorescent probe for staining amyloids in tissue sections or live mice.	Emission maximum at 520 nm Crosses the blood-brain barrier enabling in vivo staining of characteristic amyloid plaques in neurodegenerative disorders Requires a UV laser source for optimal excitation
Separation of cell lysate into insoluble and soluble fractions	Soluble proteins are dissolved with a mild detergent (e.g., Nonidet P-40, Triton X-100). Insoluble fraction is pelleted by centrifugation and lysed using strong reducing agents (e.g., 2-mercaptoethanol, SDS). Both fractions are visualized by immunoblotting.	Laborious, not suitable for large sample volumes
Correlative light and electron microscopy (CLEM) [20]	Area of interest containing a fluorescently labeled target is selected using fluorescence microscopy. Target can be analyzed at high resolution by EM.	Combines ultrastructural analysis with dynamics and improved target identification. Laborious and time-consuming Only a limited amount of cells can be examined.
Fluorescence recovery after photobleaching (FRAP) [21]	Region of a fluorescently labeled target protein is bleached with a laser. Recovery of fluorescence is recorded over time. In liquid compartments, fluorophores can diffuse, and fluorescence recovers rapidly. In solid aggregates, diffusion is not possible and fluorescence recovery is prevented.	Investigates protein mobility in living cells Laborious and time-consuming

**Table 2:** Viruses and aggregate formation. Examples of viral proteins involved in aggregate formation and their involvement in the autophagic response.

Virus	Protein	Involvement in Aggregate Formation	Involvement of Autophagy
African swine fever virus (ASFV)	-	Concentrates vDNA in aggresome-like viral factories	Not known
Baculoviruses	BmNPV polyhedrin	Co-localizes with aggregate markers	Co-localizes with LC3
Adenovirus type 5 (Ad5)	E4-11k and E1B-55k	Sequester the MRN DNA repair complex to the insoluble fraction	Re-localize components of RNA processing bodies to aggresomes
Influenza A virus (IAV)	PB1-F2	Capable of oligomerizing into amyloid fibers	Not known
Herpesviruses	M45 (MCMV)	Purified N-terminal fragment (90 aa) forms amyloid fibrils in vitro Expression in cells causes accumulation of RIPK1 and NEMO as insoluble aggregates	Recruits autophagy adapters VPS26B and TBC1D5 and degrades aggregates by autophagy
	ICP6 (HSV-1)	Induces the formation of RIPK1 aggregates	Degrades aggregates by autophagy
	ICP6 (HSV-1)	Re-localize nuclear APOBEC3 proteins to distinct structures in the cytoplasm	Not known
	BORF2 (EBV)		
	ORF61 (KSHV)		
	UL48 (HCMV)	Sequester TRIM25 in aggregate structures	Bind to 14-3-3 scaffold proteins and co-localize with p62
	BPLF1 (EBV)		
	ORF64 (KSHV)		

## References

[1] Lamark T., Johansen T. (2012). Aggrephagy: Selective Disposal of Protein Aggregates by Macroautophagy. Int. J. Cell Biol..

[2] Schmit J.D., Ghosh K., Dill K. (2011). What Drives Amyloid Molecules to Assemble into Oligomers and Fibrils?. Biophys. J..

[3] Merlini G., Bellotti V. (2003). Molecular Mechanisms of Amyloidosis. N. Engl. J. Med..

[4] Martin J.B. (1999). Molecular Basis of the Neurodegenerative Disorders. N. Engl. J. Med..

[5] Gomes E., Shorter J. (2019). The Molecular Language of Membraneless Organelles. J. Biol. Chem..

[6] Kedersha N., Anderson P. (2007). Mammalian Stress Granules and Processing Bodies. Methods Enzymol..

[7] Kedersha N., Stoecklin G., Ayodele M., Yacono P., Lykke-Andersen J., Fritzler M.J., Scheuner D., Kaufman R.J., Golan D.E., Anderson P. (2005). Stress Granules and Processing Bodies Are Dynamically Linked Sites of MRNP Remodeling. J. Cell Biol..

[8] Luo Y., Na Z., Slavoff S.A. (2018). P-Bodies: Composition, Properties, and Functions. Biochemistry.

[9] Tsai W.-C., Lloyd R.E. (2014). Cytoplasmic RNA Granules and Viral Infection. Annu. Rev. Virol..

[10] White J.P., Lloyd R.E. (2012). Regulation of Stress Granules in Virus Systems. Trends Microbiol..

[11] Kuechler E.R., Budzyńska P.M., Bernardini J.P., Gsponer J., Mayor T. (2020). Distinct Features of Stress Granule Proteins Predict Localization in Membraneless Organelles. J. Mol. Biol..

[12] Saito M., Hess D., Eglinger J., Fritsch A.W., Kreysing M., Weinert B.T., Choudhary C., Matthias P. (2019). Acetylation of Intrinsically Disordered Regions Regulates Phase Separation. Nat. Chem. Biol..

[13] Gorbenko G., Trusova V. (2011). Protein Aggregation in a Membrane Environment. Adv. Protein Chem. Struct. Biol..

[14] Bruinsma R., Pincus P. (1996). Protein Aggregation in Membranes. Curr. Opin. Solid State Mater. Sci..

[15] Parton D.L., Klingelhoefer J.W., Sansom M.S.P. (2011). Aggregation of Model Membrane Proteins, Modulated by Hydrophobic Mismatch, Membrane Curvature, and Protein Class. Biophys. J..

[16] Naiki H., Higuchi K., Hosokawa M., Takeda T. (1989). Fluorometric Determination of Amyloid Fibrils In Vitro Using the Fluorescent Dye, Thioflavine T. Anal. Biochem..

[17] Viegas M.S., Martins T.C., Seco F., do Carmo A. (2007). An Improved and Cost-Effective Methodology for the Reduction of Autofluorescence in Direct Immunofluorescence Studies on Formalin-Fixed Paraffin-Embedded Tissues. Eur. J. Histochem..

[18] Shen D., Coleman J., Chan E., Nicholson T.P., Dai L., Sheppard P.W., Patton W.F. (2011). Novel Cell- and Tissue-Based Assays for Detecting Misfolded and Aggregated Protein Accumulation within Aggresomes and Inclusion Bodies. Cell Biochem. Biophys..

[19] Skovronsky D.M., Zhang B., Kung M.P., Kung H.F., Trojanowski J.Q., Lee V.M. (2000). In Vivo Detection of Amyloid Plaques in a Mouse Model of Alzheimer’s Disease. Proc. Natl. Acad. Sci. USA.

[20] De Boer P., Hoogenboom J.P., Giepmans B.N.G. (2015). Correlated Light and Electron Microscopy: Ultrastructure Lights Up!. Nat. Methods.

[21] Ishikawa-Ankerhold H.C., Ankerhold R., Drummen G.P.C. (2012). Advanced Fluorescence Microscopy Techniques—FRAP, FLIP, FLAP, FRET and FLIM. Molecules.

[22] Balchin D., Hayer-Hartl M., Hartl F.U. (2016). In Vivo Aspects of Protein Folding and Quality Control. Science.

[23] Vembar S.S., Brodsky J.L. (2008). One Step at a Time: Endoplasmic Reticulum-Associated Degradation. Nat. Rev. Mol. Cell Biol..

[24] Bascos N.A.D., Landry S.J. (2019). A History of Molecular Chaperone Structures in the Protein Data Bank. Int. J. Mol. Sci..

[25] Vabulas R.M., Raychaudhuri S., Hayer-Hartl M., Hartl F.U. (2010). Protein Folding in the Cytoplasm and the Heat Shock Response. Cold Spring Harb. Perspect. Biol..

[26] Ran R., Lu A., Xu H., Tang Y., Sharp F.R., Lajtha A., Chan P.H. (2007). Heat-Shock Protein Regulation of Protein Folding, Protein Degradation, Protein Function, and Apoptosis. Handbook of Neurochemistry and Molecular Neurobiology: Acute Ischemic Injury and Repair in the Nervous System.

[27] Hetz C., Zhang K., Kaufman R.J. (2020). Mechanisms, Regulation and Functions of the Unfolded Protein Response. Nat. Rev. Mol. Cell Biol..

[28] Tsai Y.C., Weissman A.M. (2010). The Unfolded Protein Response, Degradation from the Endoplasmic Reticulum, and Cancer. Genes Cancer.

[29] Hwang J., Qi L. (2018). Quality Control in the Endoplasmic Reticulum: Crosstalk between ERAD and UPR Pathways. Trends Biochem. Sci..

[30] Lee S.-J., Lim H.-S., Masliah E., Lee H.-J. (2011). Protein Aggregate Spreading in Neurodegenerative Diseases: Problems and Perspectives. Neurosci. Res..

[31] Tan S., Wong E., Galluzzi L., Bravo-San Pedro J.M., Kroemer G. (2017). Chapter Fifteen—Kinetics of Protein Aggregates Disposal by Aggrephagy. Methods in Enzymology.

[32] Kopito R.R. (2000). Aggresomes, Inclusion Bodies and Protein Aggregation. Trends Cell Biol..

[33] Hochstrasser M. (1996). Ubiquitin-Dependent Protein Degradation. Annu. Rev. Genet..

[34] Hershko A., Ciechanover A. (1998). The Ubiquitin System. Annu. Rev. Biochem..

[35] Kirkin V., McEwan D.G., Novak I., Dikic I. (2009). A Role for Ubiquitin in Selective Autophagy. Mol. Cell.

[36] Fernández-Fernández M.R., Gragera M., Ochoa-Ibarrola L., Quintana-Gallardo L., Valpuesta J.M. (2017). Hsp70—A Master Regulator in Protein Degradation. FEBS Lett..

[37] Terlecky S.R., Chiang H.L., Olson T.S., Dice J.F. (1992). Protein and Peptide Binding and Stimulation of In Vitro Lysosomal Proteolysis by the 73-KDa Heat Shock Cognate Protein. J. Biol. Chem..

[38] Cuervo A.M., Dice J.F. (1996). A Receptor for the Selective Uptake and Degradation of Proteins by Lysosomes. Science.

[39] Øverbye A., Fengsrud M., Seglen P.O. (2007). Proteomic Analysis of Membrane-Associated Proteins from Rat Liver Autophagosomes. Autophagy.

[40] Bukau B., Weissman J., Horwich A. (2006). Molecular Chaperones and Protein Quality Control. Cell.

[41] Johnston J.A., Ward C.L., Kopito R.R. (1998). Aggresomes: A Cellular Response to Misfolded Proteins. J. Cell Biol..

[42] Bjørkøy G., Lamark T., Brech A., Outzen H., Perander M., Overvatn A., Stenmark H., Johansen T. (2005). P62/SQSTM1 Forms Protein Aggregates Degraded by Autophagy and Has a Protective Effect on Huntingtin-Induced Cell Death. J. Cell Biol..

[43] Kawaguchi Y., Kovacs J.J., McLaurin A., Vance J.M., Ito A., Yao T.P. (2003). The Deacetylase HDAC6 Regulates Aggresome Formation and Cell Viability in Response to Misfolded Protein Stress. Cell.

[44] Iwata A., Riley B.E., Johnston J.A., Kopito R.R. (2005). HDAC6 and Microtubules Are Required for Autophagic Degradation of Aggregated Huntingtin. J. Biol. Chem..

[45] Yao T.-P. (2010). The Role of Ubiquitin in Autophagy-Dependent Protein Aggregate Processing. Genes Cancer.

[46] Johansen T., Lamark T. (2011). Selective Autophagy Mediated by Autophagic Adapter Proteins. Autophagy.

[47] Kaganovich D., Kopito R., Frydman J. (2008). Misfolded Proteins Partition between Two Distinct Quality Control Compartments. Nature.

[48] Netherton C.L., Wileman T. (2011). Virus Factories, Double Membrane Vesicles and Viroplasm Generated in Animal Cells. Curr. Opin. Virol..

[49] Netherton C., Moffat K., Brooks E., Wileman T. (2007). A Guide to Viral Inclusions, Membrane Rearrangements, Factories, and Viroplasm Produced during Virus Replication. Adv. Virus Res..

[50] Heath C.M., Windsor M., Wileman T. (2001). Aggresomes Resemble Sites Specialized for Virus Assembly. J. Cell Biol..

[51] Novoa R.R., Calderita G., Arranz R., Fontana J., Granzow H., Risco C. (2005). Virus Factories: Associations of Cell Organelles for Viral Replication and Morphogenesis. Biol. Cell.

[52] Araujo F.D., Stracker T.H., Carson C.T., Lee D.V., Weitzman M.D. (2005). Adenovirus Type 5 E4orf3 Protein Targets the Mre11 Complex to Cytoplasmic Aggresomes. J. Virol..

[53] Liu Y., Shevchenko A., Shevchenko A., Berk A.J. (2005). Adenovirus Exploits the Cellular Aggresome Response To Accelerate Inactivation of the MRN Complex. J. Virol..

[54] Muscolino E., Schmitz R., Loroch S., Caragliano E., Schneider C., Rizzato M., Kim Y.-H., Krause E., Juranić Lisnić V., Sickmann A. (2020). Herpesviruses Induce Aggregation and Selective Autophagy of Host Signalling Proteins NEMO and RIPK1 as an Immune-Evasion Mechanism. Nat. Microbiol..

[55] Vidic J., Richard C.-A., Péchoux C., Da Costa B., Bertho N., Mazerat S., Delmas B., Chevalier C. (2016). Amyloid Assemblies of Influenza A Virus PB1-F2 Protein Damage Membrane and Induce Cytotoxicity. J. Biol. Chem..

[56] Rohrmann G.F. (2019). Baculovirus Molecular Biology.

[57] Guo Z.-J., Tao L.-X., Dong X.-Y., Yu M.-H., Tian T., Tang X.-D. (2015). Characterization of Aggregate/Aggresome Structures Formed by Polyhedrin of Bombyx Mori Nucleopolyhedrovirus. Sci. Rep..

[58] Zantema A., Fransen J.A., Davis-Olivier A., Ramaekers F.C., Vooijs G.P., DeLeys B., Van der Eb A.J. (1985). Localization of the E1B Proteins of Adenovirus 5 in Transformed Cells, as Revealed by Interaction with Monoclonal Antibodies. Virology.

[59] Greer A.E., Hearing P., Ketner G. (2011). The Adenovirus E4 11k Protein Binds and Relocalizes the Cytoplasmic P-Body Component Ddx6 to Aggresomes. Virology.

[60] Chevalier C., Al Bazzal A., Vidic J., Février V., Bourdieu C., Bouguyon E., Le Goffic R., Vautherot J.-F., Bernard J., Moudjou M. (2010). PB1-F2 Influenza A Virus Protein Adopts a Beta-Sheet Conformation and Forms Amyloid Fibers in Membrane Environments. J. Biol. Chem..

[61] Henkel M., Mitzner D., Henklein P., Meyer-Almes F.-J., Moroni A., Difrancesco M.L., Henkes L.M., Kreim M., Kast S.M., Schubert U. (2010). The Proapoptotic Influenza A Virus Protein PB1-F2 Forms a Nonselective Ion Channel. PLoS ONE.

[62] Miodek A., Vidic J., Sauriat-Dorizon H., Richard C.-A., Le Goffic R., Korri-Youssoufi H., Chevalier C. (2014). Electrochemical Detection of the Oligomerization of PB1-F2 Influenza A Virus Protein in Infected Cells. Anal. Chem..

[63] Davison A.J., Eberle R., Ehlers B., Hayward G.S., McGeoch D.J., Minson A.C., Pellett P.E., Roizman B., Studdert M.J., Thiry E. (2009). The Order Herpesvirales. Arch. Virol..

[64] Pellet P.E., Roizman B., Knipe D.M., Howley P.M. (2013). Herpesviridae. Fields Virology.

[65] Lembo D., Brune W. (2009). Tinkering with a Viral Ribonucleotide Reductase. Trends Biochem. Sci..

[66] Brune W., Ménard C., Heesemann J., Koszinowski U.H. (2001). A Ribonucleotide Reductase Homolog of Cytomegalovirus and Endothelial Cell Tropism. Science.

[67] Lembo D., Donalisio M., Hofer A., Cornaglia M., Brune W., Koszinowski U., Thelander L., Landolfo S. (2004). The Ribonucleotide Reductase R1 Homolog of Murine Cytomegalovirus Is Not a Functional Enzyme Subunit but Is Required for Pathogenesis. J. Virol..

[68] Upton J.W., Kaiser W.J., Mocarski E.S. (2008). Cytomegalovirus M45 Cell Death Suppression Requires Receptor-Interacting Protein (RIP) Homotypic Interaction Motif (RHIM)-Dependent Interaction with RIP1. J. Biol. Chem..

[69] Mack C., Sickmann A., Lembo D., Brune W. (2008). Inhibition of Proinflammatory and Innate Immune Signaling Pathways by a Cytomegalovirus RIP1-Interacting Protein. Proc. Natl. Acad. Sci. USA.

[70] Upton J.W., Kaiser W.J., Mocarski E.S. (2010). Virus Inhibition of RIP3-Dependent Necrosis. Cell Host Microbe.

[71] Johnston A., Wang Z. (2018). Necroptosis: MLKL Polymerization. J. Nat. Sci..

[72] Chen X., Li W., Ren J., Huang D., He W.-T., Song Y., Yang C., Li W., Zheng X., Chen P. (2014). Translocation of Mixed Lineage Kinase Domain-like Protein to Plasma Membrane Leads to Necrotic Cell Death. Cell Res..

[73] Upton J.W., Kaiser W.J., Mocarski E.S. (2012). DAI Complexes with RIP3 to Mediate Virus-Induced Programmed Necrosis That Is Targeted by Murine Cytomegalovirus VIRA. Cell Host Microbe.

[74] Li J., McQuade T., Siemer A.B., Napetschnig J., Moriwaki K., Hsiao Y.-S., Damko E., Moquin D., Walz T., McDermott A. (2012). The RIP1/RIP3 Necrosome Forms a Functional Amyloid Signaling Complex Required for Programmed Necrosis. Cell.

[75] Pham C.L., Shanmugam N., Strange M., O’Carroll A., Brown J.W., Sierecki E., Gambin Y., Steain M., Sunde M. (2019). Viral M45 and Necroptosis-Associated Proteins Form Heteromeric Amyloid Assemblies. EMBO Rep..

[76] Guo H., Omoto S., Harris P.A., Finger J.N., Bertin J., Gough P.J., Kaiser W.J., Mocarski E.S. (2015). Herpes Simplex Virus Suppresses Necroptosis in Human Cells. Cell Host Microbe.

[77] Huang Z., Wu S.-Q., Liang Y., Zhou X., Chen W., Li L., Wu J., Zhuang Q., Chen C., Li J. (2015). RIP1/RIP3 Binding to HSV-1 ICP6 Initiates Necroptosis to Restrict Virus Propagation in Mice. Cell Host Microbe.

[78] Dufour F., Sasseville A.M.-J., Chabaud S., Massie B., Siegel R.M., Langelier Y. (2011). The Ribonucleotide Reductase R1 Subunits of Herpes Simplex Virus Types 1 and 2 Protect Cells against TNFα- and FasL-Induced Apoptosis by Interacting with Caspase-8. Apoptosis Int. J. Program. Cell Death.

[79] Fliss P.M., Jowers T.P., Brinkmann M.M., Holstermann B., Mack C., Dickinson P., Hohenberg H., Ghazal P., Brune W. (2012). Viral Mediated Redirection of NEMO/IKKγ to Autophagosomes Curtails the Inflammatory Cascade. PLoS Pathog..

[80] Krause E., de Graaf M., Fliss P.M., Dölken L., Brune W. (2014). Murine Cytomegalovirus Virion-Associated Protein M45 Mediates Rapid NF-ΚB Activation after Infection. J. Virol..

[81] Cheng A.Z., Moraes S.N., Attarian C., Yockteng-Melgar J., Jarvis M.C., Biolatti M., Galitska G., Dell’Oste V., Frappier L., Bierle C.J. (2019). A Conserved Mechanism of APOBEC3 Relocalization by Herpesviral Ribonucleotide Reductase Large Subunits. J. Virol..

[82] Cheng A.Z., Yockteng-Melgar J., Jarvis M.C., Malik-Soni N., Borozan I., Carpenter M.A., McCann J.L., Ebrahimi D., Shaban N.M., Marcon E. (2019). Epstein-Barr Virus BORF2 Inhibits Cellular APOBEC3B to Preserve Viral Genome Integrity. Nat. Microbiol..

[83] Harris R.S., Dudley J.P. (2015). APOBECs and Virus Restriction. Virology.

[84] Harris R.S., Petersen-Mahrt S.K., Neuberger M.S. (2002). RNA Editing Enzyme APOBEC1 and Some of Its Homologs Can Act as DNA Mutators. Mol. Cell.

[85] Hultquist J.F., Lengyel J.A., Refsland E.W., LaRue R.S., Lackey L., Brown W.L., Harris R.S. (2011). Human and Rhesus APOBEC3D, APOBEC3F, APOBEC3G, and APOBEC3H Demonstrate a Conserved Capacity to Restrict Vif-Deficient HIV-1. J. Virol..

[86] Mangeat B., Turelli P., Caron G., Friedli M., Perrin L., Trono D. (2003). Broad Antiretroviral Defence by Human APOBEC3G through Lethal Editing of Nascent Reverse Transcripts. Nature.

[87] Sheehy A.M., Gaddis N.C., Choi J.D., Malim M.H. (2002). Isolation of a Human Gene That Inhibits HIV-1 Infection and Is Suppressed by the Viral Vif Protein. Nature.

[88] Zhang H., Yang B., Pomerantz R.J., Zhang C., Arunachalam S.C., Gao L. (2003). The Cytidine Deaminase CEM15 Induces Hypermutation in Newly Synthesized HIV-1 DNA. Nature.

[89] Gupta S., Ylä-Anttila P., Sandalova T., Sun R., Achour A., Masucci M.G. (2019). 14-3-3 Scaffold Proteins Mediate the Inactivation of Trim25 and Inhibition of the Type I Interferon Response by Herpesvirus Deconjugases. PLoS Pathog..

[90] Gupta S., Ylä-Anttila P., Sandalova T., Achour A., Masucci M.G. (2020). Interaction with 14-3-3 Correlates with Inactivation of the RIG-I Signalosome by Herpesvirus Ubiquitin Deconjugases. Front. Immunol..

[91] Saito S., Murata T., Kanda T., Isomura H., Narita Y., Sugimoto A., Kawashima D., Tsurumi T. (2013). Epstein-Barr Virus Deubiquitinase Downregulates TRAF6-Mediated NF-ΚB Signaling during Productive Replication. J. Virol..

[92] Van Gent M., Braem S.G.E., de Jong A., Delagic N., Peeters J.G.C., Boer I.G.J., Moynagh P.N., Kremmer E., Wiertz E.J., Ovaa H. (2014). Epstein-Barr Virus Large Tegument Protein BPLF1 Contributes to Innate Immune Evasion through Interference with Toll-like Receptor Signaling. PLoS Pathog..

[93] Ye R., Su C., Xu H., Zheng C. (2017). Herpes Simplex Virus 1 Ubiquitin-Specific Protease UL36 Abrogates NF-ΚB Activation in DNA Sensing Signal Pathway. J. Virol..

[94] Inn K.-S., Lee S.-H., Rathbun J.Y., Wong L.-Y., Toth Z., Machida K., Ou J.-H.J., Jung J.U. (2011). Inhibition of RIG-I-Mediated Signaling by Kaposi’s Sarcoma-Associated Herpesvirus-Encoded Deubiquitinase ORF64. J. Virol..

[95] Gupta S., Ylä-Anttila P., Callegari S., Tsai M.-H., Delecluse H.-J., Masucci M.G. (2018). Herpesvirus Deconjugases Inhibit the IFN Response by Promoting TRIM25 Autoubiquitination and Functional Inactivation of the RIG-I Signalosome. PLoS Pathog..

[96] Kumari P., Saha I., Narayanan A., Narayanan S., Takaoka A., Kumar N.S., Tailor P., Kumar H. (2017). Essential Role of HCMV Deubiquitinase in Promoting Oncogenesis by Targeting Anti-Viral Innate Immune Signaling Pathways. Cell Death Dis..

[97] Wang S., Wang K., Li J., Zheng C. (2013). Herpes Simplex Virus 1 Ubiquitin-Specific Protease UL36 Inhibits Beta Interferon Production by Deubiquitinating TRAF3. J. Virol..

[98] Kwon K.M., Oh S.E., Kim Y.E., Han T.-H., Ahn J.-H. (2017). Cooperative Inhibition of RIP1-Mediated NF-ΚB Signaling by Cytomegalovirus-Encoded Deubiquitinase and Inactive Homolog of Cellular Ribonucleotide Reductase Large Subunit. PLoS Pathog..

[99] Meriin A.B., Narayanan A., Meng L., Alexandrov I., Varelas X., Cissé I.I., Sherman M.Y. (2018). Hsp70–Bag3 Complex Is a Hub for Proteotoxicity-Induced Signaling That Controls Protein Aggregation. Proc. Natl. Acad. Sci. USA.

